# Strategies for equity, diversity and inclusion in geriatric healthcare professional curricula: A scoping review protocol

**DOI:** 10.1371/journal.pone.0307939

**Published:** 2024-10-03

**Authors:** Kristina M. Kokorelias, Vicky Chau, Sachindri Wijekoon, Hardeep Singh, Maurita T. Harris

**Affiliations:** 1 Department of Occupational Science & Occupational Therapy, Temerty Faculty of Medicine, University of Toronto, Toronto, Canada; 2 Toronto Rehabilitation Institute, University Health Network, Toronto, Ontario, Canada; 3 Section of Geriatrics, Department of Medicine, Sinai Health Systems and University Health Network, Toronto, Canada; 4 Rehabilitation Sciences Institute, Temerty Faculty of Medicine, University of Toronto, Toronto, Canada; 5 Divison of Geriatric Medicine, Temerty Faculty of Medicine, University of Toronto, Toronto, Canada; 6 School of Occupational Therapy, Western University, London, Canada; 7 Faculty of Liberal Arts, Wilfred Laurier University, Brantford, Canada; Xiamen University - Malaysia Campus: Xiamen University - Malaysia, MALAYSIA

## Abstract

**Introduction:**

The pursuit of Equity, Diversity and Inclusion (EDI) in healthcare education has garnered significant attention in recent years, reflecting a broader societal imperative for equitable healthcare delivery. However, existing curricula within geriatric healthcare education may not adequately address these diverse needs within their educational frameworks, inadvertently resulting in disparities in care delivery and outcomes. Within the realm of geriatric healthcare, addressing EDI is particularly crucial due to the diverse needs of older adult populations and the imperative for healthcare professionals to deliver culturally humble care. This review provides a comprehensive overview of strategies and curricular strategies, actions and/or initiatives to promote EDI within geriatric healthcare professional education.

**Methods:**

This paper presents a protocol for a forthcoming scoping review. The methodology for this scoping review adheres to the framework outlined in the Joanna Briggs Institute (JBI) Manual, encompassing four main stages: (1) formulation of a search strategy, (2) screening and selection of evidence, (3) data extraction, and (4) analysis. We will conduct a comprehensive search of peer-reviewed and empirical literature. Additionally, we will explore the reference lists of included studies to identify any relevant sources. The synthesis of findings will be conducted through a narrative approach. Reporting of the methods and results will adhere to the guidelines provided by the Preferred Reporting Items for Systematic reviews and Meta-Analyses extension for Scoping Reviews (PRISMA-ScR).

**Discussion:**

Healthcare professionals must possess the knowledge, skills, and attitudes necessary to deliver culturally humble care that respects and responds to diverse older adults’ unique needs and preferences. The review aims to fill a crucial gap in the literature by providing a comprehensive overview of strategies and curricular interventions designed to promote EDI within geriatric healthcare professional education. By mapping these strategies, actions and/or initiatives, the review seeks to identify trends, challenges, and opportunities for advancing EDI within geriatric care. The forthcoming review serves as a call to action for educators, healthcare institutions, and decision makers to prioritize EDI within geriatric healthcare education. The review identifies effective strategies and interventions for promoting EDI, providing actionable insights to inform the development of inclusive curricula, training programs, and institutional policies, which can contribute to cultivating a healthcare workforce better equipped to address the complex and evolving needs of aging populations equitably and compassionately.

## Introduction

As populations age worldwide, the number of specialized geriatric care providers who can meet the aging population’s physical and mental health needs become more important [1, 2]. A geriatric care provider is a healthcare professional specializing in the medical care and management of older adults, typically those aged 65 and above [3, 4], with age-related conditions, cognitive decline, and multiple chronic diseases [5]. Yet this specialized workforce is diminishing [6, 7]. Presently, Canada boasts roughly 376 accredited geriatricians (constituting approximately 0.004% of the total physician workforce) and 1800 certified geriatric nurses (making up about 0.0055% of registered nurses) [8]. The shortage of geriatric care providers may lead to challenges in meeting the specialized healthcare needs of older adults, with the largest disparities felt in marginalized or underserved populations [9–11]. Marginalized (or underserved) populations among older adults refer to groups within this demographic that experience barriers to accessing healthcare services and face disparities in health outcomes due to various factors such as socioeconomic status, race, ethnicity, geographic location (including rural or remote areas), disability, language barriers, or lack of adequate social support networks [9, 12–15]. These populations often encounter challenges in receiving appropriate and timely healthcare due to systemic inequities, leading to increased vulnerability and poorer health outcomes compared to more advantaged groups within the older adult population [16–19].

As geriatric care continues to be increasingly delivered to diverse populations, having a healthcare workforce that reflects this diversity and is aware of client/patient diversity is essential for person-centered and culturally humble care [20, 21]. Person-centered care “is respectful and responsive to individual patient preferences, needs, and values and ensuring that patient values guide all clinical decisions.” [20](pg. 1) Equity, Diversity and Inclusion (EDI) become critical considerations when considering how geriatric healthcare providers can address these disparities [9, 22]. Geriatric healthcare providers are increasingly tasked with ensuring that the healthcare services they provide are accessible and culturally responsive to all older adults [23–25]. In this paper, we use the term EDI to represent a framework and set of principles to promote fairness, justice, and equal opportunities for all individuals, regardless of their background, identity, or circumstances [26]. However, a recent scoping review highlighted disparities in the representation of certain marginalized groups within the context of EDI discussions, with terms linked to women and ethnic groups being more prevalent compared to terms related to disabled individuals, LGBTQ2S+ communities, and Indigenous peoples [26]; suggesting that certain groups, such as older adults with disabilities or those from LGBTQ2S+ communities- each presenting unique health needs requiring sensitivity and recognition [22], may be overlooked or underrepresented in policy frameworks. The review also highlighted the inadequacy of data and evidence informing EDI practices, particularly concerning the impact of focusing on specific marginalized groups and the intersectionality of various identities [26].

Over the past decade, there has been growing attention towards EDI in healthcare, aiming to ensure equitable access to quality care for all patients regardless of their identity [22]. Recognizing the influence of social determinants of health and intersectional identities that marginalized older adults live with is crucial for geriatric healthcare providers to deliver patient-centered and anti-oppressive care[22]. Moreover, recognizing EDI in care emphasizes the importance of comprehensive assessment and addressing cultural considerations in care [22]. By integrating EDI into the mission, vision, and values of healthcare organizations can actively embrace inclusive practices [27]. Geriatric associations such as the American Geriatrics Society (AGS) [28], Gerontological Society of America (GSA) [29] and the Geras Center for Ageing Research [30] have advocated for the inclusion of EDI principles in their curriculum and practices. While numerous educational institutions have initiated the integration of health equity curricula, these efforts frequently entail confined classroom-based activities with minimal engagement from the broader community [31]. Further examination of learning environments is required to comprehend the conditions under which learners participate in matters related to EDI [32].

A recent meta-narrative review explored how intersectionality is understood and applied in medical education, highlighting the importance of incorporating this concept into curricula and practices to advance EDI [33]. While some calls have been made to improve medical education’s grasp of intersectionality, detailed examples are scarce [32]. However, despite the recognized importance of EDI in healthcare education, there is a lack of comprehensive understanding regarding the strategies, actions and/or initiatives implemented within geriatric healthcare professional education to promote EDI. Understanding the approaches used, how they are described and evaluated in the literature, and the common trends, challenges, and opportunities associated with their implementation is essential for informing the development of effective EDI initiatives in this field [34, 35]. This is particularly important as recent geriatric medicine fellows have noted significant gaps in existing curricula on social determinants of health [11]. Additionally, while some studies describe the implementation of EDI initiatives [11, 31], there is a lack of comprehensive evaluation of their effectiveness. Furthermore, there is a need for synthesis and analysis of the existing literature to identify common trends, challenges, and opportunities in promoting EDI within geriatric healthcare education.

Expanding on the current literature, it is evident that the integration of EDI principles into geriatric healthcare education is imperative for addressing the complex needs of a diverse aging population. For example, geriatric healthcare providers must recognize that older patients may have experienced a lifetime of inequity, which can significantly impact their health outcomes and interactions with the healthcare system [36]. Studies have demonstrated that healthcare providers who receive comprehensive EDI training are better equipped to deliver culturally sensitive and person-centered care, which is crucial for improving health outcomes among marginalized older adults [37–40]. For instance, research has shown that care providers who understand and appreciate the cultural backgrounds and social determinants affecting their patients can significantly reduce health disparities and enhance the quality of care [41–43]. Despite these findings, there is a notable gap in the literature regarding the specific strategies and frameworks that effectively incorporate EDI into geriatric healthcare education. Most existing studies focus on general medical education or other healthcare fields, leaving a dearth of information on tailored approaches for geriatric care.

Furthermore, while several geriatric associations and educational institutions have begun to address EDI in their curricula, there remains a lack of comprehensive evaluation and documentation of these efforts. This gap is particularly evident in the context of intersectionality, which considers the overlapping and interdependent systems of discrimination or disadvantage experienced by individuals. Intersectionality is crucial for understanding the nuanced experiences of marginalized older adults, yet its application in geriatric education is still in its nascent stages. Recent reviews and studies have highlighted the need for more detailed examples and robust evaluations of how intersectional approaches are being integrated into medical education [33, 44–46]. By conducting this scoping review, we aim to fill these gaps by systematically examining the existing literature on EDI initiatives in geriatric healthcare education. Our goal is to identify best practices, assess the effectiveness of different strategies, and provide recommendations for future educational frameworks that can better prepare geriatric care providers to meet the diverse needs of the aging population. This review will not only contribute to the academic discourse but also offer practical insights for policymakers, educators, and healthcare providers dedicated to promoting equity and inclusivity in geriatric care.

## Methods

### Study design

This protocol was informed by the Preferred reporting items for systematic review and meta-analysis protocols (PRISMA-P) (S1 Appendix) [47]. A scoping review study design was deliberately chosen due to its suitability for investigating topics characterized by diverse literature, which may include theoretical discussions and various types of empirical studies [48]. Unlike other review methodologies aimed at addressing specific research questions or evaluating measurable outcomes of strategies, actions and/or initiatives, the primary objective of this scoping review is to systematically map and elucidate key concepts and strategies, actions and/or initiatives focusing on EDI [48, 49]. Scoping reviews serve as valuable tools for providing an overview of the landscape of existing literature, allowing researchers to gain a comprehensive understanding of the breadth and scope of the topic under investigation [48]. To guide the review’s conduct, we will follow the Joanna Briggs Institute guidance [50]. For transparency and ease of access, the protocol for this review has also been uploaded to the Open Science Framework platform (osf.io/agyvw). This review does not involve human subjects, thus obviating the need for Institutional Review Board oversight. By addressing this gap, the study aims to contribute to the development of evidence-based approaches for enhancing EDI in geriatric healthcare professional education and ultimately improving the quality of care provided to older adults from diverse backgrounds.

### Research questions

The research questions for this review are as follows:

1. What strategies, actions and/or initiatives designed to address issues of EDI in curriculum have been implemented to promote EDI within geriatric healthcare professional education?
2. How are these strategies, actions and/or initiatives described and evaluated in the literature? What are their outcomes?
3. What are the common trends, challenges, and opportunities identified in implementing EDI strategies, actions and/or initiatives within geriatric healthcare education?

### Search strategy

The search strategy will aim to identify peer-reviewed academic literature. With guidance from an expert health librarian from an academic health center, the search strategy will be developed and peer-reviewed following the Peer Review of Electronic Search Strategies (PRESS) guideline statement [51]. The strategy will first be developed in MEDLINE (Ovid), and then a comprehensive search strategy will be created for PubMed, Embase, PsycINFO, CINAHL, ERIC (Education Resources Information Center), Scopus, Social Work Abstracts, SocINDEX with Full Text, Social Sciences Citation Index, and Web of Science. The following keywords and Boolean operators will be used (see S2 Appendix):

- Equity OR Diversity OR Inclusion OR EDI
- Geriatric care OR Geriatric healthcare OR Older adults OR Elderly care
- Education OR Training OR Curriculum OR Professional development
- Healthcare providers OR Geriatricians OR Nurses OR Caregivers
- Intersectionality OR Cultural competence OR Cultural humility
- Person-centered care OR Patient-centered care

The review will consider studies published in English, with no limitations imposed on the publication dates of the included studies. The reference lists of all included documents will be examined for additional studies using citationchaser [52]. We have also formed an advisory panel of geriatric educators from two Universities in Ontario, Canada, who will be consulted to identify any relevant papers within the field that our search may have missed.

Grey literature will also be searched through OpenGrey, UpToDate, Community Research and Development Information Services, and TSpace, as well as institutional repositories of universities, healthcare institutions, and professional associations. Conference proceedings and abstract databases in the fields of geriatrics, healthcare education, and diversity inclusion will also be searched. We will also search the first 100 pages of Google, as done in other reviews [8]. Grey literature will be limited to Canada and the United States. Limiting the grey literature search to North America will help manage the search process in the context of human and time resource constraints. Focusing on a narrower geographic scope allows us to allocate our time and resources more effectively, ensuring a comprehensive search within the selected regions.

### Selection of articles

Using the PCC (Population, Concept, Context) framework by the Joanna Briggs Institute [53], the inclusion criteria for this review included:

Population:

- Learners (students, fellows) of geriatric healthcare education, including but not limited to education for physicians, nurses, social workers, and other health professionals (i.e., geriatric physical therapists, pharmacists, geriatric occupational therapists, speech-language pathologists, dieticians).

Concept:

- Articles that examine strategies, interventions, or curricula designed to promote EDI within geriatric healthcare professional education. This includes interventions to enhance cultural competence, address health inequities and improve care delivery for diverse older adult populations.

Context:

- Articles conducted in any educational setting relevant to geriatric healthcare professional education, including academic institutions, healthcare organizations, and community settings.
- No restrictions on geographical location or publication date.

After completing the search process, all identified citations will be gathered and uploaded into EndNote v.X20, where duplicate entries will be identified and removed [54]. Subsequently, the data will be imported into Covidence [55]. Titles and abstracts will be independently screened by pairs of reviewers to assess their eligibility based on the inclusion criteria for the review. The full texts of selected citations will be thoroughly evaluated against the inclusion criteria by two or more independent reviewers in Covidence, as well. Exclusion reasons for full-text documents not meeting the inclusion criteria will be documented and reported in the final review. Any disagreements arising during the selection process will be resolved through discussion. If an agreement cannot be reached, the first author (KMK) or the senior responsible author (MTH) will assign a vote. The search results and the study inclusion process will be comprehensively reported in the final review and presented using a PRISMA flow diagram [56].

### Data charting

A standardized data extraction form using Covidence will be utilized to ensure consistent information retrieval from the included studies [55]. We will extract description of the study design employed in each included article (e.g., grey literature, scoping review, systematic review, empirical study, qualitative study, quantitative study, etc.), justification provided by authors for the chosen study design, presence of research questions or aims stated in the included studies, alignment of research questions with the objectives of the scoping review, identification of specific strategies and interventions described in the literature, description of how each strategy or intervention aims to promote EDI within geriatric healthcare education, documentation of examples or case studies illustrating the implementation of these strategies and interventions, exploration of challenges or barriers encountered in implementing EDI interventions within geriatric healthcare education, documentation of any recommendations or suggestions proposed in the literature to address challenges and leverage opportunities for promoting EDI.

The form will be piloted on the first 3 articles by all members of the research team. During the pilot testing phase, the authors will independently extract data from 3 articles deemed a representative sample of studies identified for inclusion in the scoping review. This sample will be chosen to reflect the diversity and breadth of the literature relevant to our research objectives. Reviewers will follow the above mentioned predefined data extraction form. They will document any ambiguities in form instructions or discrepancies in extracted data that they encounter, providing critical feedback on their experience. Upon completion of the pilot test, we will analyze the results to identify areas requiring refinement in the data extraction forms and procedures. This analysis will focus on improving consistency among reviewers and minimizing errors in data extraction. Insights gained from the pilot test will inform adjustments to the methodology, including clarifying instructions, revising data extraction forms, and enhancing training materials for data extractors.

Throughout the data extraction process, the form will be subject to modification and refinement as needed, with any alterations documented thoroughly by the research team to aid in transparency (decision audit trail) [57, 58]. In instances where data is missing, or additional clarification is required, authors of the included studies will be contacted by the first (KMK) or senior responsible author (MTH) for supplementary information.

As a scoping review does not typically involve a formal assessment of bias risk [59], a critical appraisal of included quantitative studies will be conducted using a tool adapted from the Quality Appraisal Checklist—Quantitative Studies Reporting Correlations and Associations developed by the National Institute for Health Care Excellence (NICE) [60]. The adapted NICE tool comprises five major items focusing on the study population, selection methods, outcomes, analysis, and summary, with any irrelevant items removed to ensure applicability to the scoping review context [60]. Qualitative studies will be appraised against the Critical Appraisal Skills Programme (CASP) specifically for assessing the quality of qualitative research studies [61]. It is designed to assist researchers and reviewers in evaluating qualitative research articles’ methodological rigor, credibility, and trustworthiness [61]. Lastly, mixed-methods studies will be appraised against the Mixed Methods Appraisal Tool (MMAT) (2018 version) [62]. Commentaries, editorials and grey literature will not be appraised. The decision to add a critical appraisal was to help identify high-quality studies for potential guidance and areas for improvement in future research endeavors. Two reviewers will independently assess the methodological rigor and trustworthiness of each study. The first (KMK) and senior author (MTH) will meet with the reviewers to discuss their appraisals to resolve any discrepancies and reach a consensus on the quality assessment of each included article.

### Data analysis and reporting the results

The synthesized results will be presented through a structured approach that ensures clarity and transparency in data analysis and interpretation. Extracted data will be presented in a tabular format, outlining the study characteristics and outlines as aligned with the overarching objectives of the scoping review [59]. A descriptive analysis will be conducted based on the charted data and will also be reported as a narrative summary [59, 63]. Qualitative data extracted from the literature will undergo rigorous thematic analysis [64]. This process involves systematically identifying, organizing, and interpreting patterns, themes, and meanings within the data. Emerging themes will be derived from the study characteristics and outlines, aligning them with the overarching objectives of the scoping review. Furthermore, the literature evidence will be organized according to each research question.

For quantitative data, a comprehensive descriptive statistical analysis will be employed [65]. This approach will utilize appropriate statistical measures to quantify trends observed across the studies included in the review.

Both qualitative and quantitative findings will be synthesized and reported in a tabular format, facilitating a clear overview of the study characteristics and outcomes. A descriptive analysis will complement these tables, providing a narrative summary that synthesizes the key findings from the charted data. Furthermore, the literature evidence will be organized according to each research question, enhancing the clarity and relevance of the synthesized results to the scoping review’s objectives.

### Consultation

We will actively engage stakeholders through a pre-project advisory panel comprising geriatric healthcare provider educators from two universities in Ontario, Canada. This panel will include participants from medicine, social work and the rehabilitation sciences. We will consult the advisory council to identify relevant literature and ensure comprehensive coverage of the field, seeking their insights to refine our search and avoid overlooking critical studies. After analyzing the results, we will share a concise summary of findings with the advisory council via email. We will encourage them to contribute additional thoughts and perspectives to deepen our understanding of the data. Their feedback will be systematically integrated into the final review, influencing the interpretation of results and shaping the discussion and recommendations. Their feedback will enrich our understanding of the findings and provide diverse perspectives that we will integrate into the final discussion and recommendations of the study.

This consultative approach with our advisory panel will enhance the robustness and applicability of our research, ensuring it addresses the needs and perspectives of geriatric healthcare educators effectively.

### Timeline

The proposed timeline for the scoping review process is structured across twelve months, beginning with project initiation and planning in the first month. This phase involves forming a pre-project advisory panel comprised of geriatric healthcare provider educators from two Ontario universities. Initial planning meetings with the advisory panel will finalize research questions, objectives, and develop a comprehensive search strategy and inclusion/exclusion criteria. The literature search will span the first two months, encompassing a thorough exploration of databases and refining search strategies based on feedback from pilot testing. Months two to three will focus on screening identified studies against predefined criteria, with any conflicts resolved through consensus or third-party review. By month four, pilot testing of data extraction forms and procedures will commence, aiming to refine methodologies based on analysis of results Months four to five will be dedicated to the systematic extraction of data from selected studies, ensuring consistency and accuracy among data extractors. This phase will include addressing any discrepancies through consensus to maintain robust data quality. In months six to seven, data synthesis will occur, involving thematic analysis for qualitative data and statistical analysis for quantitative data where applicable. Writing and drafting activities will take place during months eight to nine, encompassing the drafting of the scoping review manuscript, incorporation of feedback from the advisory panel, and development of discussion and recommendations sections. Month ten will focus on the review and finalization of the manuscript, including revisions based on peer feedback, finalization of tables, figures, and preparation of the final report and executive summary. Month eleven will involve submission of the manuscript to the target journal or publication venue, preparation of presentations or summaries for dissemination, and sharing findings with stakeholders and the academic community. Post-review activities in month twelve will entail responding to any peer review comments, publishing the scoping review results, and archiving data and documentation for transparency and future reference.

## Discussion

The scoping review protocol outlined in this paper addresses the need to understand interventions to support EDI within geriatric healthcare professional education. By systematically mapping and elucidating key concepts and interventions focusing on EDI within this context, the review aims to contribute to the development of evidence-based approaches for informing future curricula. By synthesizing and analyzing the existing literature, the review will identify common trends, challenges, and opportunities associated with the implementation of EDI initiatives in geriatric healthcare professional education. In addition, by identifying successful strategies and interventions, educators and policymakers can design curriculum enhancements and training programs that better address the needs of older adults from marginalized or underserved populations; ultimately improving the quality of care provided to older adults from diverse backgrounds. While not a primary objective of the study, this scoping review is also anticipated to highlight areas where additional research is needed [59].

This study is strengthened by its rigourous and systematic approach. The review protocol is informed by established guidelines such as the Preferred Reporting Items for Systematic Review and Meta-Analysis Protocols (PRISMA-P) [47] and the Joanna Briggs Institute guidance [66, 67]. By encompassing diverse literature, including theoretical discussions and various types of empirical studies, the review is anticipated to provide a comprehensive overview of the topic. In addition, the review considers studies from various disciplines relevant to geriatric healthcare education, including medicine, nursing, social work, and allied health professions.

Despite its potential contributions, this scoping review has several limitations that warrant consideration. Firstly, as with any literature review, the search strategy employed in this review may not capture all relevant studies. Despite efforts to develop a comprehensive search strategy and consult with experts in the field, some relevant literature may be overlooked. Additionally, limiting the search to studies published in English may result in the exclusion of relevant research published in other languages. Despite these limitations, this scoping review protocol represents an important step towards understanding and enhancing EDI within geriatric healthcare professional education. These limitations will be transparently discussed in the review, highlighting efforts taken to mitigate biases and offering suggestions for future research directions.

## Conclusion

The paper presents a protocol for a forthcoming scoping review to identify studies examining strategies, interventions, or curricula designed to promote EDI within geriatric healthcare education settings. The integration of EDI principles into geriatric healthcare education is essential for fostering a healthcare workforce equipped to deliver person-centered and anti-oppressive care [22]. While efforts have been made to incorporate EDI into educational curricula, there remains a lack of comprehensive understanding regarding the strategies and interventions implemented within geriatric healthcare professional education. This comprehensive review will contribute to developing evidence-based approaches for enhancing EDI in geriatric healthcare professional education, ultimately improving the quality of care provided to older adults from diverse backgrounds.

## Supporting information

S1 Appendix PRISMA-P (Preferred Reporting Items for Systematic review and Meta-Analysis Protocols) 2015 checklist: Recommended items to address in a systematic review protocol*. (DOCX)

S2 Appendix Medline search. (DOCX)

## References

[1] O’Neill G, Barry P. Training physicians in geriatric care: Responding to critical need: National Academy on an Aging Society; 2003.

[2] Medicine AGSCWGotTFotFoG. Caring for older Americans: The future of geriatric medicine. Journal of the American Geriatrics Society. 2005;53(S6):S245–S56. doi: 10.1111/j.1532-5415.2005.53350.x 15963180

[3] Warshaw GA, Bragg EJ. The essential components of quality geriatric care. Generations. 2016;40(1):28–37.

[4] Campbell JY, Durso SC, Brandt LE, Finucane TE, Abadir PM. The unknown profession: a geriatrician. Journal of the American Geriatrics Society. 2013;61(3):447–9. doi: 10.1111/jgs.12115 23350990 PMC3793249

[5] Leipzig RM, Sauvigné K, Granville LJ, Harper GM, Kirk LM, Levine SA, et al. What is a geriatrician? American Geriatrics Society and Association of Directors of Geriatric Academic Programs end‐of‐training entrustable professional activities for geriatric medicine. Journal of the American Geriatrics Society. 2014;62(5):924–9. doi: 10.1111/jgs.12825 24749846

[6] Fisher JM, Garside M, Hunt K, Lo N. Geriatric medicine workforce planning: a giant geriatric problem or has the tide turned? Clinical Medicine. 2014;14(2):102. doi: 10.7861/clinmedicine.14-2-102 24715117 PMC4953277

[7] Kovner CT, Mezey M, Harrington C. Who cares for older adults? Workforce implications of an aging society. Health affairs. 2002;21(5):78–89. doi: 10.1377/hlthaff.21.5.78 12224911

[8] Krause KE, Kokorelias KM, Sinha SK. A systematic and qualitative analysis of geriatric models of care for rural and remote populations. Rural and Remote Health. 2022;22(3):1–11. 10.22605/RRH7486 35975281

[9] Veltman A, La Rose T. Marginalized geriatric patients. Geriatric Psychiatry: A Case-Based Textbook. 2018:629–43.

[10] Santos P, Faughnan K, Prost C, Tschampl CA. Systemic barriers to care coordination for marginalized and vulnerable populations. Journal of Social Distress and Homelessness. 2023;32(2):234–47.

[11] Robertson ML, Mushero N, Demers L, Goroncy A, Chippendale R. Inequities in the care of older adults: Identifying education gaps in geriatric medicine fellowship. Gerontology & Geriatrics Education. 2023;44(2):254–60. doi: 10.1080/02701960.2022.2047037 35272580

[12] Kokorelias KM, Wasilewski MB, Flanagan A, Zhabokritsky A, Singh H, Dove E, et al. Co-creating socio-culturally-appropriate virtual geriatric care for older adults living with HIV: a community-based participatory, intersectional protocol. International Journal of Qualitative Methods. 2023;22:16094069231205189.

[13] Co Toronto. Factsheet on Equity-Seeking Groups 2018.

[14] Arms T, Keilman LJ, Peraza-Smith GB. Culturally Informed Mental Health Care of Marginalized Older Adults. Advances in Family Practice Nursing. 2022;4(1):37–53.

[15] D’cruz M, Banerjee D. ‘An invisible human rights crisis’: The marginalization of older adults during the COVID-19 pandemic–An advocacy review. Psychiatry research. 2020;292:113369. doi: 10.1016/j.psychres.2020.113369 32795754 PMC7397988

[16] Kehoe MacLeod K, Flores KN, Chandra K. Identifying facilitators and barriers to integrated and equitable care for community-dwelling older adults with high emergency department use from historically marginalized groups. International Journal for Equity in Health. 2023;22(1):97. doi: 10.1186/s12939-023-01900-y 37208757 PMC10198019

[17] Mulumba M, Nantaba J, Brolan CE, Ruano AL, Brooker K, Hammonds R. Perceptions and experiences of access to public healthcare by people with disabilities and older people in Uganda. International journal for equity in health. 2014;13:1–9. 25928896 10.1186/s12939-014-0076-4 PMC4188877

[18] Cheng SM, Catallo C. Healthy at Home: an integrated health and social care initiative for vulnerable and marginalized older adults in Toronto. Journal of Integrated Care. 2024.

[19] Martin JL. Organizational strategies for addressing disparities among marginalized older adults: University of Denver; 2015.

[20] Borkan JM, Culhane-Pera KA. Towards cultural humility in healthcare for culturally diverse Rhode Island. Rhode Island Medical Journal. 2008;91(12):361. 19170310

[21] Lekas H-M, Pahl K, Fuller Lewis C. Rethinking cultural competence: Shifting to cultural humility. Health services insights. 2020;13:1178632920970580. doi: 10.1177/1178632920970580 33424230 PMC7756036

[22] Veltman A, La Rose T. Equity, Diversity, and Inclusion in the Care of Older Adults. Geriatric Psychiatry: A Case-Based Textbook: Springer; 2024. p. 861–77.

[23] Dixon JS, Mather MA, Ready RE, Madore MR. Culturally responsive psychological assessment with racially and ethnically diverse older adults. Psychological assessment. 2023;35(1):82. doi: 10.1037/pas0001189 36227304

[24] Kurpas D, Gwyther H, Szwamel K, Shaw RL, D’Avanzo B, Holland CA, et al. Patient-centred access to health care: a framework analysis of the care interface for frail older adults. BMC geriatrics. 2018;18:1–17. 30419817 10.1186/s12877-018-0960-7 PMC6233263

[25] Anderson LM, Scrimshaw SC, Fullilove MT, Fielding JE, Normand J, Services TFoCP. Culturally competent healthcare systems: A systematic review. American journal of preventive medicine. 2003;24(3):68–79. 12668199 10.1016/s0749-3797(02)00657-8

[26] Wolbring G, Nguyen A. Equity/equality, diversity and inclusion, and other EDI phrases and EDI policy frameworks: A scoping review. Trends in Higher Education. 2023;2(1):168–237.

[27] Johnson K, Ali H, Bryan J, Kernick A, Kitty D, Primavesi R, et al. Leadership for change: how medical associations are working toward equity, diversity, and inclusion. Canadian Journal of Emergency Medicine. 2023;25(4):274–7. doi: 10.1007/s43678-023-00452-y 36725784 PMC9891743

[28] Rhodes RL, Saliba D, Brangman SA, Lundebjerg NE, Ouslander JG, Smith AK. Change is coming: Efforts to enhance diversity, equity, and inclusion within the Journal of the American Geriatrics Society. Wiley Online Library; 2022. p. 2751–3. doi: 10.1111/jgs.18015 36056693

[29] GSA. The Gerontological Society of America (GSA) Policy on Diversity, Equity, and Inclusion 2023. Available from: https://www.geron.org/About-GSA/Commitment-to-Diversity-Equity-Inclusion.

[30] Research GCfA. Equity, Diversity and Inclusion. Available from: https://www.gerascentre.ca/equity-diversity-and-inclusion/.

[31] Denizard-Thompson N, Palakshappa D, Vallevand A, Kundu D, Brooks A, DiGiacobbe G, et al. Association of a health equity curriculum with medical students’ knowledge of social determinants of health and confidence in working with underserved populations. JAMA network open. 2021;4(3):e210297–e. doi: 10.1001/jamanetworkopen.2021.0297 33646312 PMC7921901

[32] Gilliam CA, Grow HM, Homer P, Mullett TA, Willgerodt M, Kunze-Garcia S, et al. “The curriculum brings equity to the forefront”: Pediatric residents’ perspectives and experiences in a longitudinal EDI curriculum. Journal of the National Medical Association. 2023;115(1):3–14. doi: 10.1016/j.jnma.2022.12.009 36599745

[33] Rehman M, Santhanam D, Sukhera J. Intersectionality in medical education: a meta-narrative review. Perspectives on medical education. 2023;12(1):517. doi: 10.5334/pme.1161 37954042 PMC10637289

[34] Severance J, Gordon B, Lindberg B. Policy Series: Building Momentum for Diversity, Equity, and Inclusion in Geriatrics and Gerontology Education. Innovation in Aging. 2021;5(Supplement_1):89–.

[35] Schnable TG. Application of the Social Determinants of Health to Geriatric Patients by Nurse Practitioner Students: Marquette University; 2023.

[36] Health U. Principles of EDI 2024. Available from: https://www.uclahealth.org/departments/medicine/geriatrics/about-us/principles-edi.

[37] Booysen LA, Gill P. Creating a culture of inclusion through diversity and equity. Management and Leadership Skills for Medical Faculty and Healthcare Executives: A Practical Handbook. 2020:135–44.

[38] Etowa J, Debs-Ivall S. Leadership and Organizational Commitment to Ethno-Cultural Diversity in Healthcare. Journal of Ethnographic & Qualitative Research. 2017;11(4).

[39] Kersey-Matusiak G. Delivering culturally competent nursing care: Working with diverse and vulnerable populations: Springer Publishing Company; 2024.

[40] Weissman JS, Betancourt J, Campbell EG, Park ER, Kim M, Clarridge B, et al. Resident physicians’ preparedness to provide cross-cultural care. Jama. 2005;294(9):1058–67. doi: 10.1001/jama.294.9.1058 16145026

[41] Sciences NAo Health BoG, Health CoEHPtAtSDo. A framework for educating health professionals to address the social determinants of health. 2016. 27854400

[42] Brach C, Fraserirector I. Can cultural competency reduce racial and ethnic health disparities? A review and conceptual model. Medical care research and review. 2000;57(1_suppl):181–217. doi: 10.1177/1077558700057001S09 11092163 PMC5091811

[43] Like RC, Barrett TJ, Moon J. Educating physicians to provide culturally competent, patient-centered care. Perspectives. 2008;7(2):10–20.

[44] Bochatay N, Bajwa NM, Ju M, Appelbaum NP, van Schaik SM. Towards equitable learning environments for medical education: bias and the intersection of social identities. Medical education. 2022;56(1):82–90. doi: 10.1111/medu.14602 34309905

[45] Harari L, Lee C. Intersectionality in quantitative health disparities research: A systematic review of challenges and limitations in empirical studies. Social science & medicine. 2021;277:113876. doi: 10.1016/j.socscimed.2021.113876 33866085 PMC8119321

[46] Anglim C. Intersectionality as a Critical Framework for Medical Ethics Education. Journal of the Society of Christian Ethics. 2023;43(1):93–109.

[47] Moher D, Shamseer L, Clarke M, Ghersi D, Liberati A, Petticrew M, et al. Preferred reporting items for systematic review and meta-analysis protocols (PRISMA-P) 2015 statement. Systematic reviews. 2015;4:1–9. doi: 10.1186/2046-4053-4-1 25554246 PMC4320440

[48] Colquhoun HL, Levac D, O’Brien KK, Straus S, Tricco AC, Perrier L, et al. Scoping reviews: time for clarity in definition, methods, and reporting. Journal of clinical epidemiology. 2014;67(12):1291–4. doi: 10.1016/j.jclinepi.2014.03.013 25034198

[49] Tricco AC, Lillie E, Zarin W, O’brien K, Colquhoun H, Kastner M, et al. A scoping review on the conduct and reporting of scoping reviews. BMC medical research methodology. 2016;16:1–10. 26857112 10.1186/s12874-016-0116-4 PMC4746911

[50] Hong QN, Pluye P, Bujold M, Wassef M. Convergent and sequential synthesis designs: implications for conducting and reporting systematic reviews of qualitative and quantitative evidence. Systematic reviews. 2017;6:1–14. 28335799 10.1186/s13643-017-0454-2 PMC5364694

[51] McGowan J, Sampson M, Salzwedel DM, Cogo E, Foerster V, Lefebvre C. PRESS peer review of electronic search strategies: 2015 guideline statement. Journal of clinical epidemiology. 2016;75:40–6. 27005575 10.1016/j.jclinepi.2016.01.021

[52] Haddaway NR, Grainger MJ, Gray CT. Citationchaser: A tool for transparent and efficient forward and backward citation chasing in systematic searching. Research Synthesis Methods. 2022;13(4):533–45. doi: 10.1002/jrsm.1563 35472127

[53] Pollock D, Peters MDJ, Khalil H, McInerney P, Alexander L, Tricco AC, et al. Recommendations for the extraction, analysis, and presentation of results in scoping reviews. JBI evidence synthesis. 2023;21(3):520–32. doi: 10.11124/JBIES-22-00123 36081365

[54] Bramer WM, Giustini D, de Jonge GB, Holland L, Bekhuis T. De-duplication of database search results for systematic reviews in EndNote. Journal of the Medical Library Association: JMLA. 2016;104(3):240. 27366130 10.3163/1536-5050.104.3.014 PMC4915647

[55] Babineau J. Product review: Covidence (systematic review software). Journal of the Canadian Health Libraries Association/Journal de l’Association des bibliothèques de la santé du Canada. 2014;35(2):68–71.

[56] Tricco AC, Lillie E, Zarin W, O’Brien KK, Colquhoun H, Levac D, et al. PRISMA extension for scoping reviews (PRISMA-ScR): checklist and explanation. Annals of internal medicine. 2018;169(7):467–73. doi: 10.7326/M18-0850 30178033

[57] Levac D, Colquhoun H, O’Brien KK. Scoping studies: advancing the methodology. Implementation science. 2010;5:1–9. 20854677 10.1186/1748-5908-5-69 PMC2954944

[58] Carcary M. The research audit trail: Methodological guidance for application in practice. Electronic Journal of Business Research Methods. 2020;18(2):pp166‑77-pp‑77.

[59] Arksey H O’Malley L Scoping studies: towards a methodological framework. International journal of social research methodology. 2005;8(1):19–32.

[60] Excellence NIfC. Quality appraisal checklist–quantitative studies reporting correlations and associations. Methods for the development of NICE public health guidance. 2012.

[61] Long HA, French DP, Brooks JM. Optimising the value of the critical appraisal skills programme (CASP) tool for quality appraisal in qualitative evidence synthesis. Research Methods in Medicine & Health Sciences. 2020;1(1):31–42.

[62] Hong QN. Revision of the Mixed Methods Appraisal Tool (MMAT): A mixed methods study: McGill University (Canada); 2018.

[63] Pautasso M. The structure and conduct of a narrative literature review. A guide to the scientific career: Virtues, communication, research and academic writing. 2019:299–310.

[64] Braun V, Clarke V. Using thematic analysis in psychology. Qualitative research in psychology. 2006;3(2):77–101.

[65] Mishra P, Pandey CM, Singh U, Gupta A, Sahu C, Keshri A. Descriptive statistics and normality tests for statistical data. Annals of cardiac anaesthesia. 2019;22(1):67–72. doi: 10.4103/aca.ACA_157_18 30648682 PMC6350423

[66] Peters MD, Marnie C, Tricco AC, Pollock D, Munn Z, Alexander L, et al. Updated methodological guidance for the conduct of scoping reviews. JBI evidence implementation. 2021;19(1):3–10. doi: 10.1097/XEB.0000000000000277 33570328

[67] Porritt K, Gomersall J, Lockwood C. JBI’s systematic reviews: study selection and critical appraisal. AJN The American Journal of Nursing. 2014;114(6):47–52. doi: 10.1097/01.NAJ.0000450430.97383.64 24869584

