# Supplementary material for: Strategies for equity, diversity and inclusion in geriatric healthcare professional curricula: A scoping review protocol
Source: PLoS One. 2024 Oct 3;19(10):e0307939. doi: 10.1371/journal.pone.0307939 (PMC11449309; doi:10.1371/journal.pone.0307939)
Supplement: S2 Appendix — (DOCX) [file pone.0307939.s002.docx]

OVID Medline

Ovid MEDLINE(R) ALL <1946 to May 09, 2024>

1 Geriatrics/ 31659

2 Geriatricians/ 303

3 (Geriatric* or Gerontolog*).tw,kw,kf. 88640

4 1 or 2 or 3 95757

5 Education, Medical/ 62001

6 Education, Medical, Graduate/ 34023

7 Education, Medical, Undergraduate/ 27653

8 Education, Medical, Continuing/ 25656

9 "Internship and residency"/ 62272

10 "Fellowships and Scholarships"/ 9960

11 Teaching Rounds/ 1534

12 Curriculum/ 89562

13 (Education or Educator? or Teacher? or Trainee* or Trained or Training or Resident* or Residency or Residencies or Intern* or Student* or learner* or Fellow?).tw,kf,kw. 2828100

14 (Teaching Rounds or Fellowship?).tw,kf,kw. 16580

15 (Curriculum or Curricula).tw,kf,kw. 74429

16 ((Geriatric* adj1 Nurses) or (Geriatric* adj1 Nursing) or (Geriatric* adj1 Physical Therapist*) or (Geriatric* adj1 Pharmacist*) or (Geriatric* adj1 Occupational Therapist*) or (Geriatric* adj2 Pathologist*) or (Geriatric* adj1 Dietician*) or (Gerontolog* adj1 Nurses) or (Gerontolog* adj1 Nursing) or (Gerontolog* adj1 Physical Therapist*) or (Gerontolog* adj1 Pharmacist*) or (Gerontolog* adj1 Occupational Therapist*) or (Gerontolog* adj2 Pathologist*) or (Gerontolog* adj1 Dietician*)).tw,kf,kw. 2948

17 5 or 6 or 7 or 8 or 9 or 10 or 11 or 12 or 13 or 14 or 15 or 16 2908254

18 Diversity, Equity, Inclusion/ 383

19 Cultural Diversity/ 13097

20 Cultural Competency/ 6686

21 (EDID or EDI or DEI or Decolonization*).tw,kf,kw. 9301

22 (Healthcare adj1 Equity).tw,kw,kf. 239

23 (Diversity or Diverse or Diversities or Equity or Equality or Inclusion* or Inclusivity or Inclusiveness or Belonging).tw,kf,kw. 1227998

24 ((Cultural adj1 competenc*) or (Cultural adj1 sensitivit*) or (Cultural adj1 humilit*)).tw,kf,kw. 7319

25 intersectionality.tw,kf,kw. 2878

26 18 or 19 or 20 or 21 or 22 or 23 or 24 or 25 1252592

27 4 and 17 and 26 1349
